# Role of Depression and Anxiety Disorders in Takotsubo Syndrome: The Psychiatric Side of Broken Heart

**DOI:** 10.7759/cureus.10400

**Published:** 2020-09-11

**Authors:** Federico Oliveri, Harshit K Goud, Lubna Mohammed, Zainab Mehkari, Moiz Javed, Aldanah Althwanay, Farah Ahsan, Ian H Rutkofsky

**Affiliations:** 1 Cardiology, California Institute of Behavioral Neurosciences & Psychology, Fairfield, USA; 2 Internal Medicine, California Institute of Behavioral Neurosciences & Psychology, Fairfield, USA; 3 Obstetrics & Gynaecology, California Institute of Behavioral Neurosciences & Psychology, Fairfield, USA; 4 Psychiatry, California Institute of Behavioral Neurosciences & Psychology, Fairfield, USA

**Keywords:** takotsubo syndrome, psychiatric disorders, anxiety disorders, depression

## Abstract

Takotsubo syndrome (TTS), also called broken heart syndrome, is an acute and transient cardiac wall motion abnormality of the left ventricle. The patient prototype is a post-menopausal woman with myocardial infarction-like symptoms (angina pectoris, breathlessness, palpitations, etc.) who has experienced sudden emotional or physical stress. Although prognosis is generally considered relatively benign, both complications and recurrence rates are not insignificant. Pathophysiological mechanisms underlying TTS are not entirely understood, but the sympathetic system over-activity has a leading role. Moreover, since emotional stress frequently triggers TTS and since precedent diagnosis of psychiatric disorders sometimes coexists, the psychological response to stress could be another potential therapeutic target. Indeed, this article aims to explore the association between underlying depression and anxiety disorders and TTS, as well as to find ideal therapeutic options useful to treat and prevent TTS. Thus in our review, we considered case reports, case-control studies, and review articles from PubMed. Papers dealing with Takotsubo syndrome and anxiety disorder or depression were selected. We included papers published since 2010 and whose abstract was in English. We concluded that anxiety disorders, but not depression, are associated with a higher occurrence of TTS. There is a link between anxiety, TTS, and inflammation leading to increased sympathetic activity. Nevertheless, patients with pre-admission psychiatric disorders have a higher risk of recurrent TTS. Importantly, the use of selective serotonin reuptake inhibitors (SSRIs) could be a potential therapeutic aid in preventing TTS's recurrence in selected patients.

## Introduction and background

Takotsubo syndrome (TTS), also called apical ballooning syndrome (ABS), broken heart syndrome, or stress cardiomyopathy, is a syndrome characterized by transient regional systolic dysfunction of the left ventricle (LV), resembling myocardial infarction (MI), but in the absence of angiographic evidence of acute plaque rupture or hemodynamically significant obstructive coronary artery disease (CAD) [1]. Although there are different subtypes of regional systolic disfunction, the “classical” anatomical pattern of TTS is characterized by left ventricle regional wall motion abnormality with apical and circumferential mid-ventricular hypokinesia and basal hyperkinesia. At end-systole, the left ventricle typically resembles the ‘Takotsubo’ (Japanese name used to indicate an octopus pot) [2], with a narrow neck and larger lower portion, giving the appearance of apical ballooning [3,4]. Two out of 10,000 hospitalizations (0.02%) in the United States are considered caused by TTS [5]. The majority of patients (89-91%) are elderly post-menopausal women (66-80 years of age), while the overall mean age is 66.9 ± 30.7 years and most patients (59.6%) are ≥65 years old [4-6]. Indeed the typical patient with primary TTS is a post-menopausal woman who has experienced severe, sudden emotional or physical stress [4]. It can happen in a background of high personal stress or, in some cases, in patients with a previous diagnosis of psychiatric disorders [5,7]. However, the mortality rate is higher in men (8.4% vs. 3.6%), probably reflecting the higher frequency of underlying severe critical illness (36.6% in men vs. 26.8% in women) [6]. Patients with TTS typically present with acute angina pectoris, breathlessness, palpitations, but also ventricular tachyarrhythmias and/or cardiogenic shock may be present [4]. Diagnosis of Takotsubo syndrome is usually made following the Mayo Clinic diagnostic criteria, all four of which are required [8]: I. Reversible (transient) left ventricular systolic (LV) dysfunction (dyskinesis, hypokinesis, akinesis). Left ventricle regional wall motion abnormality extends beyond a single epicardial vascular distribution; II. Absence of significant obstructive coronary artery disease (CAD) or angiographic evidence of acute plaque rupture. If CAD is present, the diagnosis of TTS can still be made if the wall motion abnormalities extend beyond a single epicardial vascular distribution; III. New ECG abnormalities (either T-wave inversion or ST-segment elevation) or modest elevation in cardiac troponin; IV. Absence of myocarditis or pheochromocytoma.

Although Takotsubo syndrome is generally considered having a relatively benign prognosis with rapid recovery of LV function, newer evidence suggests TTS is a more acute severe cardiac disorder with a variety of complications in ∼52% of patients [9-11]. Systolic heart failure is the most common complication in the acute phase of Takotsubo syndrome, occurring in 12-45% of cases [10-12]. Ventricular arrhythmias occur in 4-9% of patients during the acute phase of the syndrome, causing cardiac arrest in 4-6% of cases [13-15]. The in-hospital mortality rate is 2-5%, mainly due to ventricular fibrillation or refractory cardiogenic shock [5,6,9-11]. The pathophysiology of Takotsubo syndrome is complex and not fully understood but catecholamines appear to play a pivotal role as the trigger is often sudden unexpected stress [4]. Five-year recurrence rates of 5-22% have been reported, with the second episode occurring three months to 10 years after the first [4,16]. Preventive therapy of recurrence has yet to be found. Intuitively, beta-blockers may provide some protection against future catecholamine surges. However, recurrences have been reported in patients taking beta-blockers, and meta-analysis found no impact of these medications on the risk of recurrence [17]. Given the high frequency of stressful triggers in cases of TTS [4], the psychological response to stress could be another potential therapeutic target. Indeed, since both mortality and recurrence rates are significant [4,6,16], this review aims to explore the possible association between underlying psychiatric disorders and TTS. Nevertheless, by better understanding the pathophysiological mechanisms underlying TTS, potential useful therapeutic targets could be found to reduce both the onset and recurrence of Takotsubo syndrome.

## Review

Method

Research Process

The research procedure was conducted in two steps. Firstly we have searched articles about risk factors, pathophysiology, treatment, and recurrence prevention of Takotsubo syndrome; then, we combined Takotsubo and psychiatric disorders, selecting articles that deal with depression and anxiety disorders in patients with coexisting TTS. We used PubMed and Google Scholar as primary electronic databases to find materials. Keywords used for our process were: Takotsubo syndrome, psychiatric disorders, anxiety disorders, depression. Results are presented as follow (Table 1):

**Table 1:** Keywords and Number of Articles Found

KEYWORD/COMBINATION OF KEYWORDS	NUMBER OF RESULTS
Psychiatric disorders	472 689
Depression	213 902
Anxiety	123 969
Takotsubo syndrome	3 923
Takotsubo syndrome and psychiatric disorders	155
Takotsubo syndrome and depression	147
Takotsubo syndrome and anxiety	72

Inclusion and Exclusion Criteria

Case reports, case-control studies, and review articles were considered in our review. Papers dealing with Takotsubo syndrome and anxiety disorder or depression were included. We selected publications whose abstract was in English, while papers published before 2010 were not considered. No geographic considerations were given.

Discussion

Pre-Existing Psychiatric Disorders and TTS

To date, both depression and anxiety disorders are associated with increased cardiovascular disease, events, and death [18-20]. Specifically, anxiety is perhaps considered the most critical risk factor for cardiovascular disease [21]. Less evidence regarding the possible association between psychiatric disorders, specifically anxiety disorder and depression, and Takotsubo syndrome is available. A retrospective study compared TTS patients taken by the International Takotsubo Registry with 455 sex- and age-matched patients with a diagnosis of acute coronary syndrome (ACS). This study was conducted to investigate epidemiological characteristics, clinical features, prognostic predictors, and outcomes of TTS. The analysis showed that total psychiatric disorders, both acute and chronic, were statistically more frequent in patients with TTS than those with ACS; past or chronic anxiety disorders were statistically more prevalent in the TTS group than the ACS group; and there was no statistically prevalence difference in acute anxiety disorders between the two groups [22] (Table 2).

**Table 2:** Comparison of the Prevalence of Psychiatric Disorders in Patients With TTS and ACS TTS: Takotsubo syndrome, ACS: acute coronary syndrome

PSYCHIATRIC DISORDER	TTS (n)	TTS (%)	ACS (n)	ACS (%)	P-VALUE
Total (acute+chronic)	191	42,3	64	14,3	<0,001
Acute psychiatric disorder	57	12,6	6	1,3	<0,001
Acute anxiety disorder	4	0,9	0	0,0	0,120
Past or chronic psychiatric disorders	165	36,6	61	13,6	<0,001
Past or chronic anxiety disorders	45	10,0	4	0,9	<0,001

It should be noted that the authors did not analyze “depression” alone as a separate condition [22]. Therefore the possibility that depression had been more prevalent in one group or another cannot be affirmed or excluded.

In another retrospective case-control study, the authors assessed past psychiatric history preceding the episode of ABS in 25 TTS patients by comparing them with two group controls. The first control group was based on 25 age- and sex-matched patients with previous ST-elevation myocardial infarction (STEMI), while the second one included 50 age- and sex-matched patients from the general population (GP) [23]. The results showed that chronic anxiety disorders were statistically more frequent in patients with Takotsubo syndrome group than the other groups, and depression did not occur with statistically significant differences between the three groups (Table 3).

**Table 3:** Comparison of the Prevalence of Psychiatric Disorders in Patients With TTS, STEMI, and GP TTS: Takotsubo syndrome, STEMI: ST-elevation myocardial infarction, GP: general population

PSYCHIATRIC DISORDER	TTS (%)	STEMI (%)	GP (%)	P-VALUE
Anxiety or depression	68	36	30	<0,050
Chronic Anxiety disorders	56	12	18	<0,001
Depression	48	28	22	>0,050

So the previous study confirmed the higher prevalence of pre-morbid chronic anxiety disorders in TTS. Still, it does not highlight a statistically significant difference in depression disorders between the three groups. This study’s strength is the use of two control groups (STEMI and GP), while its limitations are small sample size and non-multi-centric [23].

Another case-control study based on women with TTS confirms that compared to myocardial infarction (MI) and general healthy (GH) controls, the pre-admission prevalence of anxiety disorders is statistically more prevalent in patients who had developed TTS than in the other two groups (24.4% in TTS, 9.4% in MI, and 0 in GH with p=0.007). Moreover, this study has also shown that depression was not associated with the occurrence of TTS [24]. Since more than 90% of patients diagnosed with TTS are women [4-6], the sample based on women only is a relative limitation. A further study conducted on a huge number of patients with previous diagnoses of TTS (n=24,701) confirmed the higher prevalence of anxiety disorders in this group compared to MI and orthopedic controls [25].

On the other hand, it should also be noted that some authors have reported conflicting data. For example, in a case-control study, 50 TTS patients and sex- and age-matched STEMI controls were asked to complete, during the hospitalization, a survey that measured the self-reported trait anxiety. A high-anxiety trait was a common finding, but there was not a statistically significant difference between the two groups [26]. Nevertheless, it should also be considered that the survey was submitted during the hospital stay, possibly being influenced by hospitalization-related anxiety. Another case-control study based on 19 patients with TTS and sex-, age-, and residence-matched controls has shown that only major depressive disorder (MDD) had a significantly higher prevalence in Takotsubo patients than controls [27]. The main limitations of the previous two above studies were the small sample size.

Indeed, by comparing the aforementioned studies, we are comfortable stating that anxiety, more precisely chronic anxiety disorders, is related to the occurrence of Takotsubo syndrome [22-25]. On the other hand, depression does not appear to be more prevalent in TTS patients [23,24].

TTS Triggers in Patients With and Without Pre-Admission Anxiety Disorders

The typical patient with primary TTS is a post-menopausal woman who has experienced severe, unexpected emotional or physical stress [4,28]. Some authors have recently described that in patients with pre-admission anxiety disorders, TTS was predominantly triggered by exclusively emotional stress. In this study, triggering events were compared in 58 TTS patients with (group1) and without (group2) pre-existing anxiety disorders. The results showed that in patients with a past medical history positive for anxiety disorders, TTS was mainly triggered by a emotional stress; in patients without pre-existing anxiety disorders, undetermined event are more likely to trigger TTS; and physical triggers are more frequently associated with the second group but without statistically significant difference (Table 4) [29].

**Table 4:** Triggers in Patients With and Without Pre-Admission Anxiety Disorders

TRIGGER	PRE-ADMISSION ANXIETY DISORDER PATIENTS	PRE-ADMISSION WITHOUT ANXIETY DISORDERS PATIENTS	P-VALUE
Emotional stress	74%	30%	0,001
Physical stress	16%	37%	0,070
Undetermined	33%	10%	0,027

Table 5 mentions case-reports of patients with a diagnosis of Takotsubo syndrome and coexisting anxiety disorders, most of which were triggered by emotional, stressful events.

**Table 5:** Case Report of Patient With TTS and Concomitant Anxiety Disorderstriggers in Patients With and Without Pre-Admission Anxiety Disorders TTS: Takotsubo syndrome

AUTHORS	CASES	PRE-ADMISSION ASSOCIATED PSYCHIATRIC DISORDER
Toni et al. 2019 [30]	A 65 years old woman with a history of two previous episodes of TTS and medically treated anxiety had a sudden death during minor oral surgery. A post-mortem examination showed apical biventricular ballooning and no coronary artery disease, compatible with another episode of TTS.	Chronic anxiety not better defined.
Vergel et al. 2016 [31]	A 65-year-old woman with a history of an untreated generalized anxiety disorder presented typical symptoms of acute coronary syndrome (ACS) after she had experienced the violent death of her son. The followed coronary angiogram did not show significant obstructions. In the end, the diagnosis of TTS was made.	Chronic anxiety (Generalized anxiety disorder).
Elsayed et al. 2019 [32]	A 43-year-old female patient presented to the psychiatric outpatient clinic after experiencing severe work-related bullying. She complained of acute chest pain in a background of depressed mood, low energy, anhedonia, generalized anxiety, and sleep difficulties, present for several weeks. The initial ECG was unremarkable, but serum troponin was elevated. The patient was transferred to the cardiology department, and coronary angiogram excluded an ACS. In the end, apical ballooning and left ventricular dysfunction, compatible with TTS, was found.	-Chronic anxiety (Generalized anxiety disorder); -Acute social stress (bullying); -Major depressive disorder.
Chadha 2020 [33]	An 85-year-old female presented to the ER with sudden onset chest pain. The patient admitted being extremely stressed due to the current COVID-19 pandemic. However, labs and imaging excluded a possible COVID-19 infection. The ECG showed an abnormal ST pattern in leads V1-V3, and her initial Troponin T was also elevated. An urgent cardiac catheterization was made, which excluded hemodynamically significant coronary artery disease (CAD). Moreover, left ventriculogram (LVG) revealed basal hyperkinesis and apical ballooning. The ejection fraction checked by Echocardiogram was initially 35%, while five days later the Echo showed complete recovery of the LV systolic function.	Anxiety not better defined.
Champ-Rigot, et al. 2011 [34]	A 49-year old woman with a history of anxiety disorders presented to a hospital because of an acute anxiety attack. She admitted recently to be very emotionally stressed. The first ECG was quite normal, but troponin-I was elevated. She was transferred to the cardiac ICU. Cardiac catheterization did not show any hemodynamically significant coronary occlusions. LVG highlighted hypo-contraction limited to mid-segments with hyper-contractility of the apex. Echo performed 72h after cardiac angiography was unremarkable.	Chronic anxiety disorder with superimposed acute anxiety disorder.
Ikram et al. 2016 [35]	A 46-year-old woman with a history of motor vehicle accident complicated by subdural hematoma (SDH) and subarachnoid hemorrhage (SAH) was admitted to the hospital to laryngoscopy and eventual tracheal stenosis treatment to be performed under general anesthesia. Since her previous admission, she had been in therapy with alprazolam, bupropion, citalopram, and tizanidine because of organic post-traumatic anxiety and depression (caused by SDH and SAH). On induction of anesthesia, she developed acute left ventricle failure secondary to “atypical” TTS.	Post-traumatic anxiety and depression with superimposed physical stress.

Takotsubo and Anxiety Disorders: Pathophysiologic Connections

Although the pathophysiology of TTS is not fully understood, much evidence has been documented. At the very beginning, spasms of coronary arteries were identified as TTS's underlying mechanism, although this hypothesis was later refuted [28]. However, it is well known that brain neurons that mediate vasoconstriction innervate coronary microcirculation, supports the concept that regional myocardial stunning among patients with TTS may be of neurogenic origin [22]. By the time, numerous studies demonstrated increased sympathetic activity, and consequent catecholamine excess, in TTS patients [27,36]. The higher epinephrine levels stimulate the β2-adrenoceptor coupling switch from membranous Gs to Gi proteins, with a consequent negative inotropic effect. It leads to cardiac protection because the “Gs->Gi switch” limits the degree of acute myocardial injury in response to the catecholamine excess [36,37]. Moreover, it was proven that the highest density of β-adrenergic receptors in the human heart is the apex [36]. Thus, this different anatomical distribution, coupled with the catecholamine over-release, could explain the characteristic “apical ballooning” of the majority cases of TTS. Patients affected by depression or anxiety disorders also show sympathetic overactivity in response to physical and emotional stress. Psycho-neuro-endocrinological activation of the autonomic nervous system is expressed in two-stage responses [38]: I. acute response mediated by the activation of the central nervous systemadrenal-medulla axis, leading to catecholamine release in the adrenal medulla; II. chronic response caused by activation of hypothalamus-pituitary-adrenal axis secondary to stressors, leading to cortisol release from the adrenal cortex. Indeed, the sympathetic system hyperactivity occurring in both TTS and anxiety disorders may in part explain the link between these conditions.

Furthermore, some authors have described a connection between TTS and inflammatory responses of the myocardium. In murine-based experiments, a predominant myocardial M1 macrophage infiltration (proinflammatory tissue destructive) was found in TTS, and no significant switch to M2 macrophages appeared (anti-inflammatory and profibrotic function) [39]. It has also been demonstrated that cytokines, which increase during inflammation state, indirectly increase the sympathetic output from central and peripheral autonomic nervous system nerve fibers and directly affect ion channels of cardiomyocytes. Specifically, interleukin (IL)-1β and IL-6 enhance cardiac calcium channels, while tumor necrosis factor-α (TNF-α) reduced the expression of potassium channels on myocardial cells [40]. The final effect of these ion current changes leads to electrocardiographic QTc prolongation, frequently found in TTS [4]. On the other hand, it has been verified that serum cytokine concentration is higher in many patients suffering from anxiety disorders [41].

**Figure 1:**
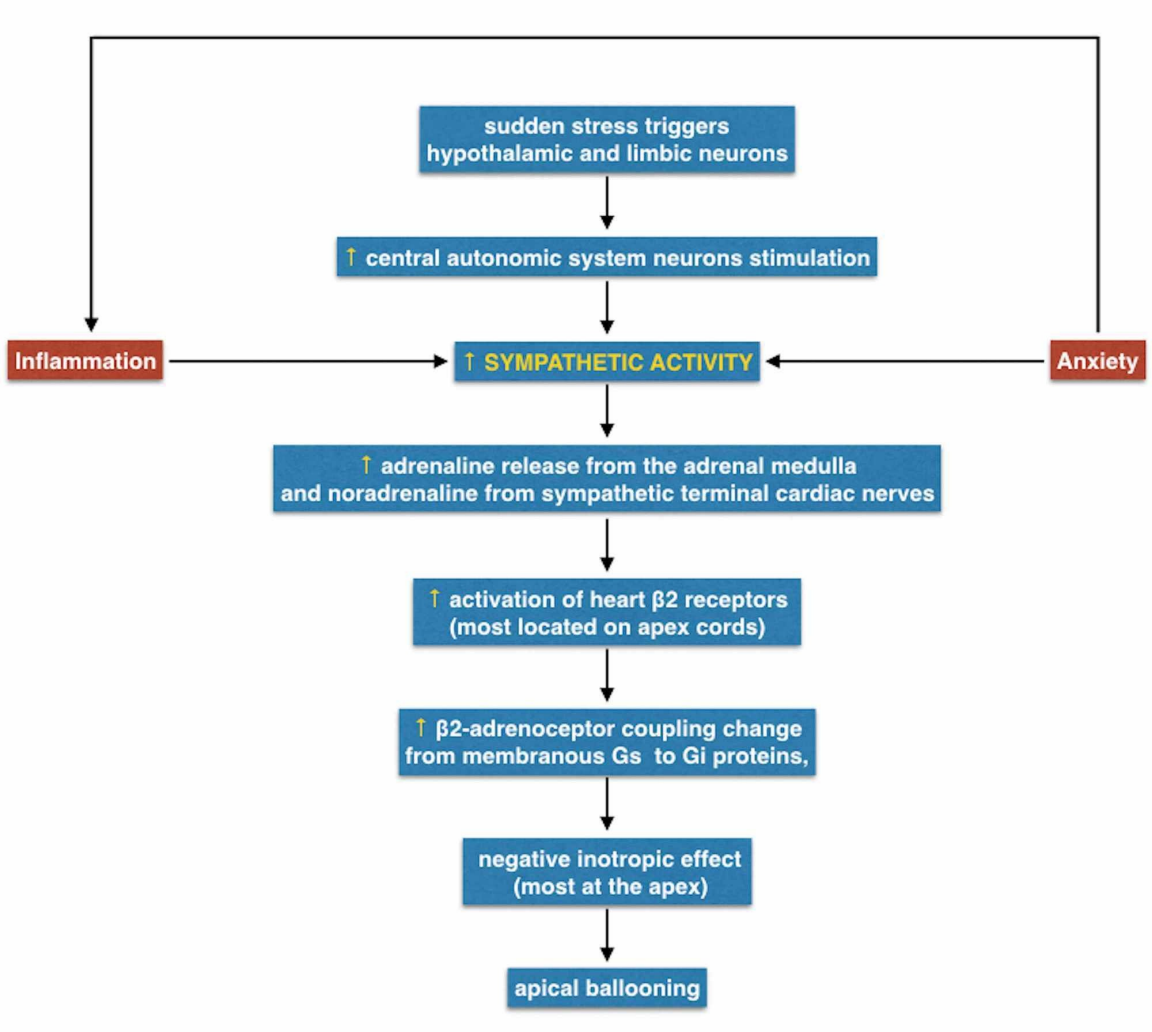
Takotsubo and Anxiety Disorders: Pathophysiologic Connections

We propose an integrated model with the aim of integrating the different studies concerning the pathophysiology of TTS together (Figure 1):

Psychoactive Drugs

Since a connection between anxiety disorders and TTS occurrence has been reported [22-25], it would seem conceivable that, by adequately treating anxiety disorders, we could reduce the incidence of TTS. Nevertheless, data available in the literature do not show univocal results. For example, in a case report of a 67-year-old woman with a past medical history of anxiety and major depression disorders in treatment with clonazepam and fluoxetine (a selective serotonin reuptake inhibitor or SSRI), the tapering of her medication may have triggered an episode of TTS. Since she felt good and her mood had been stable at the time, her doctor decided to lower the dosage of anxiolytics. After three days, she developed chest pain, so she decided to go to the ER. After hospitalization, a diagnosis of TTS was made. Thus, the authors of this study hypothesized a protective role of anxiolytics in the development of TTS [42].

Another case report partially supports the above result. In this study, a postmenopausal woman with a history of TTS in preventive treatment with beta-blockers and ACE-inhibitors had a recurrence of Takotsubo syndrome six months from the previous diagnosis. She was subsequently discharged with sertraline (an SSRI) plus cognitive-behavioral therapy (CBT) without recurrent episodes during the following months. Indeed, the authors sustained that the combination of SSRI-CBT therapy should be a possible therapeutic aid in preventing TTS recurrence [43]. However, it should also be considered that in a significant retrospective multi-centered study, it has been evaluated that antidepressants, specifically SSRIs, were taken more frequently at admission in TTS patients than in ACS controls (respectively 17.1% vs 6.9% and 10.3% vs 3.4%; p-value <0.001) [22]. Nevertheless, this result can be explained by looking at the greater prevalence of psychiatric diseases, primarily anxiety, in patients with TTS in this study. Moreover, to date, there have been no clear cases of SSRIs as triggers of TTS [44].

On the other hand, some authors consider the use of serotonin-norepinephrine reuptake inhibitors (SNRIs) could trigger TTS [45,46]. In a case report, a 52-year-old female, after being put on venlafaxine because of depression, developed TTS a few weeks later. She was discharged without venlafaxine, and, at the six-week follow-up visit, left ventricle kinesis and motion were unremarkable [45]. Again, in another case report, the use of duloxetine (another SNRI) seems to have been the trigger for a TTS episode [46]. The patient of this study, a 59-year-old woman with a history of depression, developed TTS after one week from the upper-titration of duloxetine. During the hospitalization, duloxetine therapy was discontinued. After one month from the acute event, the patient's left ventricular function recovered entirely without reoccurrence [46]. It may be plausible that the increased postsynaptic concentration of serotonin and norepinephrine caused by SNRIs could trigger TTS episodes. However, given the limited extension of these studies and the limited follow-up of the patients enrolled, it could be premature to suggest discontinuation of venlafaxine, duloxetine, or other SNRIs until new evidence confirms the results of the above studies.

Recurrence and Complications

Five-year recurrence rates occur in about 5-22%, with the second episode occurring three months to 10 years after the first [4,16]. However, few studies have investigated the role of pre-existing psychiatric disorders as possible promoters of TTS recurrence. In a study conducted on 306 patients with TTS, one-third of whom had pre-existing psychiatric disorders, the TTS recurrence rate at follow-up was approximately 7%. Notably, patients with pre-existing psychiatric illness were at higher risk of recurrent TTS. Nevertheless, from the same study, pre-existing psychiatric disorders were not associated with a more prevalent 30-day or long-term mortality [47].

Acute in-hospital complications are more prevalent in TTS patients with pre-existing psychiatric disorders. To demonstrate this, a retrospective analysis conducted on 455 TTS highlighted that presence of pre-existing psychiatric disorders as well as physical triggers, acute neurologic disorders, an elevated first troponin (more than 10 times the upper limit), and a reduced left ventricular ejection fraction of less than 45% were independent predictors for acute in-hospital complications. Authors included death from any cause, need for catecholamine use, cardiogenic shock, use of invasive or noninvasive ventilation, or cardiopulmonary resuscitation as acute in-hospital complications [22]. In another retrospective study, the authors calculated the ejection fraction (EF) recovery time in 36 TTS patients. The median recovery of EF was 25 days. Then they divided “early recoverers” patients whose EF returned to normal in less than or equal to 25 days (group 1) and “late recoverers” whose recovery needed more than 25 days (group 2). Demographic and clinical factors were compared between the groups. Generalized anxiety disorder was seen more commonly in the group with early recovery [48]. Thus long-term complications seem no to be affected by pre-existing psychiatric disorders [47,48].

Limitations

Our review does not take into account articles published before 2010. Furthermore, we selected publications whose abstract was written in English. Unfortunately, many of the papers analyzed are based on case reports or retrospective case-controls with small sample sizes. While searching the literature, there were no specific clinical trials that deal with psychiatric disorders/anxiety disorders and Takotsubo syndrome. Moreover, by thoroughly reading each article involved, the diagnosis of anxiety disorders or depression was not established by using the same diagnostic criteria.

## Conclusions

As many cases of TTS are triggered by emotional stress, we wondered if there was a higher prevalence of psychiatric disorders in these patients to exploit, in the absence of specific therapy, new therapeutic targets usable to prevent TTS onset and recurrence. The pre-admission prevalence of anxiety disorders is more prevalent in patients who developed TTS than in the healthy population and STEMI/ACS controls. On the other hand, we are confident to affirm that depression is not associated with a higher occurrence of TTS.

There is a robust pathophysiological interconnection between anxiety, inflammation, and TTS, leading to an increase in sympathetic activity. Patients with pre-admission psychiatric disorders have a higher risk of recurrent TTS and are more predisposed to suffer from acute in-hospital complications. The use of SSRIs, possibly in combination with CBT therapy, could be a potential therapeutic aid in preventing TTS's recurrence in selected patients. Since our review showed that anxiety disorders promote the onset and recurrence of TTS, a multidisciplinary approach, including psychiatrists or psychologists, may be highly desirable to provide the best care while reducing costs due to intra-hospital complications. Moreover, our review lays the foundations for possible new studies. For example, having documented a potential "protective role" of SSRIs, It would be useful to conduct a clinical trial where a group of patients with established anxiety disorders and TTS would receive beta-blockers (controls). In contrast, another group would be treated with beta-blocker plus SSRI. 
